# Artificial Intelligence for ADRD Caregivers: A Systematic Literature Review

**DOI:** 10.1093/geroni/igaa057.2804

**Published:** 2020-12-16

**Authors:** Bo Xie, Cui Tao, Juan Li, Robin Hilsabeck, Alyssa Aguirre

**Affiliations:** 1 The University of Texas at Austin, Austin, Texas, United States; 2 School of Biomedical Informatics, The University of Texas Health Science Center at Houston, Houston, Texas, United States; 3 Naval Medical University, Shanghai, China; 4 University of Texas, Austin, Texas, United States; 5 Dell Medical School - University of Texas, Austin, Texas, United States

## Abstract

Artificial intelligence (AI) may improve the care for persons with Alzheimer’s disease and related dementias (ADRD) and caregivers’ quality of life. To examine existing research on this topic, we searched 5 publication databases using keywords related to Alzheimer’s, dementia, caregiver, and artificial intelligence, and found 113 relevant results. We then screened the titles, abstracts, and full texts, and excluded studies not including family caregivers, not involving providing information to users, screening/diagnosis of dementia, caregiver anxiety/burnout, or lacking empirical data. Thirty results remained. These studies included technologies such as robots to facilitate social engagement and assist with daily activities. Most were qualitative or cross-sectional with small samples. These studies generally reported positive outcomes in favor of AI tools but levels of evidence were low. These findings suggest the need for systematic designs that can generate high levels of evidence for the feasibility and efficacy of AI-based interventions for ADRD caregivers.

